# Dihydromyricetin Attenuates Diabetic Cardiomyopathy by Inhibiting Oxidative Stress, Inflammation and Necroptosis via Sirtuin 3 Activation

**DOI:** 10.3390/antiox12010200

**Published:** 2023-01-15

**Authors:** Yun Chen, Yangyang Zheng, Ruixiang Chen, Jieru Shen, Shuping Zhang, Yunhui Gu, Jiahai Shi, Guoliang Meng

**Affiliations:** 1 Department of Pharmacology, School of Pharmacy, Nantong University, Nantong 226001, China; 2 Nantong Key Laboratory of Translational Medicine in Cardiothoracic Diseases, and Research Institution of Translational Medicine in Cardiothoracic Diseases, Nantong University, Nantong 226001, China

**Keywords:** dihydromyricetin, diabetic cardiomyopathy, oxidative stress, necroptosis, sirtuin 3

## Abstract

Dihydromyricetin (DHY), the main flavonoid component in *Ampelopsis grossedentata*, has important benefits for health. The present study aimed to investigate the exact effects and possible mechanisms of DHY on diabetic cardiomyopathy (DCM). Male C57BL/6 mice and sirtuin 3 (SIRT3) knockout (SIRT3-KO) mice were injected with streptozotocin (STZ) to induce a diabetic model. Two weeks later, DHY (250 mg/kg) or carboxymethylcellulose (CMC) were administrated once daily by gavage for twelve weeks. We found that DHY alleviated fasting blood glucose (FBG) and triglyceride (TG) as well as glycosylated hemoglobin (HbA1c) levels; increased fasting insulin (FINS); improved cardiac dysfunction; ameliorated myocardial hypertrophy, fibrosis and injury; suppressed oxidative stress, inflammasome and necroptosis; but improved SIRT3 expression in STZ-induced mice. Neonatal rat cardiomyocytes were pre-treated with DHY (80 μM) with or without high glucose (HG) stimulation. The results showed that DHY attenuated cell damage but improved SIRT3 expression and inhibited oxidative stress, inflammasome and necroptosis in cardiomyocytes with high glucose stimulation. Moreover, the above protective effects of DHY on DCM were unavailable in SIRT3-KO mice, implying a promising medical potential of DHY for DCM treatment. In sum, DHY improved cardiac dysfunction; ameliorated myocardial hypertrophy, fibrosis and injury; and suppressed oxidative stress, inflammation and necroptosis via SIRT3 activation in STZ-induced diabetic mice, suggesting DHY may serve as a candidate for an agent to attenuate diabetic cardiomyopathy.

## 1. Introduction

Currently, the burdens of diabetes and related complications have become a major threat to public health. Diabetic cardiomyopathy (DCM), one of the most devastating complications resulting from chronic diabetes, is a cardiac pathological condition characterized by abnormal cardiac morphology and cardiac dysfunction in the absence of other cardiovascular factors, such as hypertension, valvular disease and coronary artery disease. The combination of lifestyle management and blood glucose regulation through hypoglycemic drugs or insulin replacement therapy is the main strategy to inhibit the incidence of DCM [1]. However, there is no specific treatment targeting DCM up to the moment.

Dihydromyricetin (DHY), a flavonoid isolated from *Ampelopsis grossedentata*, exhibited several important pharmacological benefits, including anti-inflammatory, antioxidant, hepatoprotective and cardioprotective effects. It has been reported that DHY treatment alleviated doxorubicin-induced cardiotoxicity by inhibiting the NLR family pyrin domain-containing protein 3 (NLRP3) inflammasome through sirtuin 1 activation [2] and protected against atherogenesis by increasing endothelial nitric oxide production [3]. Our previous study verified that DHY attenuated transverse aortic constriction induced myocardial hypertrophy [4], alleviated cardiac fibroblasts proliferation and cardiomyocyte hypertrophy by angiotensin II (Ang II) stimulation [5,6], improved endothelial dysfunction in diabetic mice [7] and inhibited cellular cholesterol accumulation in oxidized low-density lipoprotein (ox-LDL)-stimulated macrophages [8]. However, whether DHY could alleviate DCM and especially the detailed mechanism remains unknown.

It has been well established that DCM is attributed to various factors, including hyperglycemia, insulin disorder, inflammation, oxidative stress, cardiomyocyte apoptosis and others [9,10,11]. High glucose may directly mediate proinflammatory factors secretion and perpetuate the inflammatory process, causing cardiomyocyte hypertrophy, mitochondrial dysfunction, endoplasmic reticulum stress and death, fibroblast proliferation, collagen production and then promoting the development of DCM [12]. Furthermore, hyperglycemia induces excessive generation of reactive oxygen species (ROS), which may also initiate inflammatory response and trigger progressive mitochondrial damage and cardiomyocyte apoptosis in diabetic heart, finally leading to cardiac dysfunction, remodeling and heart failure [13]. Recent studies have reported that necroptosis is also involved in DCM [14,15,16]. Necroptosis is a highly inflammatory form of cell death, which is triggered by receptor interacting kinase 1 (RIPK1), then binds to receptor interacting kinase 3 (RIPK3) and subsequently recruits and phosphorylates mixed lineage kinase domain-like protein (MLKL). Among them, RIKP3 is the key indicator of necroptosis. Accumulating studies demonstrated that necroptosis is a cell death connecting oxidative stress, inflammation and cardiovascular diseases [15,17]. However, whether DHY suppresses necroptosis during DCM is unclear.

Sirtuin 3 (SIRT3) is a deacetylase widely expressed in the heart, which modulates proteins to regulate energy metabolism or apoptosis to protect against oxidative stress [18,19,20]. Thus, targeting SIRT3 has been proposed as a promising therapeutic strategy for ameliorating cardiac disturbances. Our previous study found that SIRT3 deficiency aggravated hyperglycemia-induced mitochondrial damage, increased ROS accumulation, promoted necroptosis, activated NLRP3 inflammasome and ultimately exacerbated DCM in the mice [14]. However, whether SIRT3 is the critical target for DHY in alleviating DCM is not clear, which remains to be further determined.

The present study aims to investigate the effects and the detailed mechanism of DHY on DCM. It is beneficial to provide a novel candidate for the prevention and treatment of diabetic cardiomyopathy.

## 2. Materials and Methods

### 2.1. Animal Treatments

Male 8-week-old C57BL/6 mice and SIRT3-knockout (SIRT3-KO) mice with 129S1/SvImJ background were intraperitoneally injected with streptozotocin (STZ, 60 mg/kg/day, Sigma-Aldrich, St. Louis, MO, USA) for 5 consecutive days after an overnight fast of 12 h. Mice in the control group were injected with the same amount of citrate buffer. Fasting blood glucose (FBG) was detected with a OneTouch glucometer after fasting for 8 h. Mice with FBG above 16.7 mmol/L are diagnosed as diabetic mice. Two weeks after STZ injection, DHY (250 mg/kg, Standard Center of China, Beijing, China) dissolved in carboxymethylcellulose (CMC, 0.5%, Sinopharm Chemical Reagent Co., Ltd., Shanghai, China) was administrated once daily by gavage for twelve weeks [4,7]. CMC with equal amount were given as control. All animals (n = 6 in each group) were subjected to echocardiography and then sacrificed for subsequent experiments.

### 2.2. Neonatal Rat Cardiomyocyte Culture and Treatment

Primary culture of cardiomyocytes was prepared from Sprague-Dawley rats (1–3 days old) as described before [21]. After culturing in Dulbecco’s modified eagle medium (DMEM, Wisent Inc., Montreal, QC, Canada) with 10% fetal bovine serum (FBS, Wisent Inc., Montreal, QC, Canada) for 48 h, the cells were pre-treated with DHY (80 μM) for 4 h [6], followed by normal glucose (control, 5.5 mM) or high glucose (HG, 33.3 mM) stimulation for 48 h.

### 2.3. Measurement of Triglyceride (TG), Glycosylated Hemoglobin (HbA1c) and Fasting Insulin (FINS) Levels

The measurement of TG in serum and HbA1c in plasma was performed respectively by TG and HbA1c Assay Kit (Jiancheng, Nanjing, China) according to the manufacturer’s instructions. The FINS level was detected with the insulin radioimmunoassay kit (Beijing Furui Bio-Tec Co., Ltd., Beijing, China).

### 2.4. Echocardiography

The mice were anesthetized with 2.5% isoflurane and maintained with 1.5% isoflurane. Anesthesia was monitored by the lack of reflex to toe pinching. The transthoracic echocardiography was conducted with the Vevo 2100 echocardiography system (Visualsonics, Toronoto, ON, Canada) as described before [14]. Ejection fraction (EF) and fraction shortening (FS) were measured to evaluate cardiac systolic function. The pulse wave of Doppler E wave and A wave velocity of the mitral valve were taken, and the E/A ratio was calculated to assess cardiac diastolic function.

### 2.5. Histological Analysis

The myocardium of one-third of the left ventricle was fixed in 4% paraformaldehyde, embedded in paraffin and then sliced to sections with thickness of 5 μm. The sections were subjected to hematoxylin eosin (HE) staining to assess myocardial hypertrophy. Sirus red as well as Masson’s staining were used to evaluate myocardial fibrosis.

### 2.6. Measurement of Lactate Dehydrogenase (LDH) and Adenosine Triphosphate (ATP)

Cardiomyocytes injury was evaluated with LDH levels in the serum and cell culture medium, as well as the ATP levels in the myocardium and cardiomyocytes. They were determined according to the instructions of the commercial kits, respectively (Beyotime, Shanghai, China).

### 2.7. Assessment of Oxidative Stress

The oxidative stress level was assessed with dihydroethidium (DHE) and MitoSOX staining. Myocardial tissue sections of left ventricle or cardiomyocytes were incubated with DHE (Beyotime, Shanghai, China) to detect the production of superoxide. MitoSOX (YEASEN, Shanghai, China) and Mito-tracker (Beyotime, Shanghai, China) were incubated to detect the level of mitochondrial superoxide. To stain the nuclei, 4′,6-diamidino-2-phenylindole (DAPI, Beyotime, Shanghai, China) was used. The fluorescence was detected with a laser confocal microscope (Leica, Wetzlar, Germany).

### 2.8. Terminal Deoxynucleotidyl Transferase-Mediated dUTP Nick End-Labelling (TUNEL) Assay

The apoptosis was assessed using a TUNEL kit (Beyotime, Shanghai, China) according to the manufacturer’s instructions. Paraffin sections were incubated with TUNEL, and images were captured using optical microscopy. The cardiomyocytes were stained with fluorescein-dUTP in the presence of terminal deoxynucleotidyl transferase (TdT) after fixation with immunofluorescence fixative solution. Then the nuclei were stained with DAPI and observed with laser confocal microscope.

### 2.9. Quantitaive Real-Time PCR

Total RNA was extracted from myocardium about 2 mm × 2 mm and cardiomyocytes using TRIzol reagent (Takara, Kyoto, Japan). RNA samples (1000 ng) were subjected to complementary DNA (cDNA) synthesis by PrimeScriptTM RT Master Mix kit (Takara, Kyoto, Japan). Then, quantitative PCR was performed using the SYBR Green reagents on ABI 7500 RT-PCR system (Carlsbad, CA, USA). The primer sequences (Sangon Biotech Co., Ltd., Shanghai, China) are listed as follows: mice atrial natriuretic peptide (ANP, NM_008725), sense 5′-GAGAAGATGCCGGTAGAAGA-3′ and antisense 5′ -AAG-CACTGCCGTCTCTCAGA-3′; mice brain natriuretic peptide (BNP, NM_008726), sense 5′-CTGCTGGAGCTGATAAGAGA-3′ and antisense 5′ -TGCCCAAAG-CAGCTTGAGAT-3′; rat SIRT3 (NM_001106313), sense 5′-GAGGTTCTTGCTGCATGTGGTTG-3′ and antisense 5′-AGTTTCCCGCTGCACAAGGTC-3′; 18S, sense 5′-AGTCCCTGCCCTTTGTACACA-3′ and antisense 5′-CGATCCGAGGGCCTCACTA-3. 18S was used as a housekeeping gene and the mRNA levels were normalized to the control samples.

### 2.10. Western Blotting

Total protein content was harvested from frozen myocardium about 3 mm × 3 mm and cardiomyocytes using the lysis buffer (Beyotime, Shanghai, China) containing 1% phenylmethylsulfonyl fluoride. Proteins (20–50 μg) were separated by sodium dodecyl sulfate-polyacrylamide gel electrophoresis (SDS-PAGE) and transferred to polyvinylidene fluoride membrane (PVDF) membranes (Millipore, Billerica, MA, USA). The membranes were blocked with 5% non-fat milk, and then incubated with primary antibodies against anti-p-MLKL, anti-SIRT3 (1:1000, Cell Signaling Technology, Danvers, MA, USA); anti-RIPK3 (1:1000, Novusbio, Littleton, CO, USA); anti-MLKL (1:1000), anti-interleukin-1β (IL-1β, 1:1000), anti-NLRP3 (1:1000, Abcam, Cambridge, UK); anti-β-tubulin (1:3000, CMCTAG, Milwaukee, WI, USA) or anti-GAPDH (1:5000, Sevicebio, Wuhan, China). After overnight incubation, the membranes were incubated with the secondary antibodies (1:5000, ZSbio, Beijing, China). The blots were visualized using enhanced chemiluminescence (ECL, Thermo Fisher Scientific Inc., Rockford, IL, USA).

### 2.11. Immunofuorescence

After blocking with blocking solution containing 0.5% Triton X-100, the cardiomyocytes were incubated with primary anti-NLRP3 (1:200), or anti-caspase 1 (1:200) antibodies. Subsequently, the cells were incubated with IgG conjugated with Cy3 or Alexa Fluor 488 (Beyotime, Shanghai, China). The nuclei were stained with DAPI. The cardiomyocytes were observed with laser confocal microscope.

### 2.12. Statistical Analysis

All data are expressed as mean ± standard error of the mean (SEM), and analysis was performed by one-way analysis of variance and a Bonferroni post-hoc test. Values of *p* less than 0.05 are considered to be statistically significant.

## 3. Results

### 3.1. DHY Alleviated FBG, TG, HbA1c Level and Increased FINS in STZ-Induced Mice

On the third day after STZ injection, C57BL/6 mice showed a gradual increase in food and water intake, as well as urine volume, and had manifestations such as body weight loss and lusterless, dry or messy hair, as compared with mice in the control group. The symptoms of mice after DHY administration were somewhat relieved.

**Figure 1:**
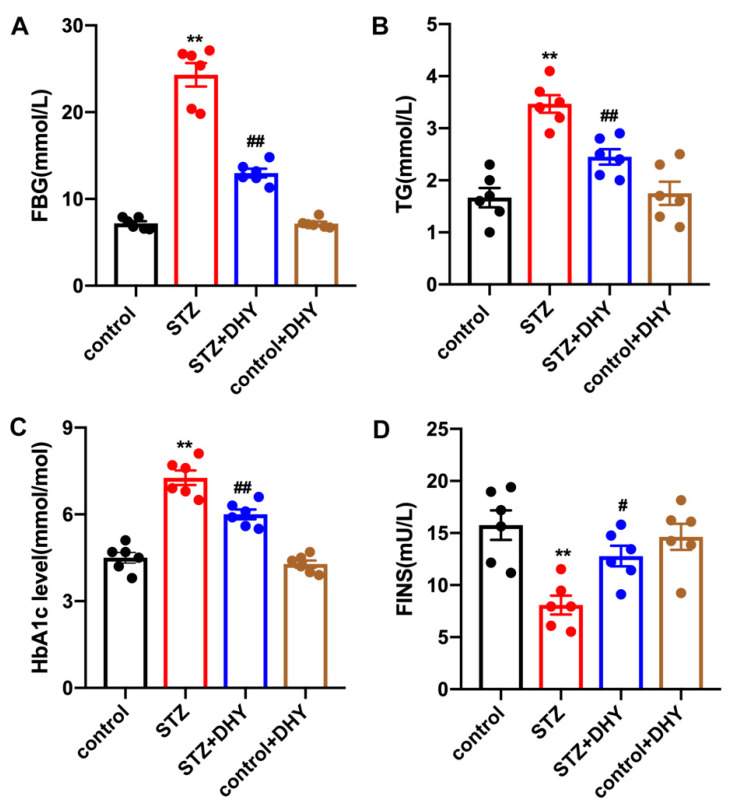
DHY alleviated FBG, TG and HbA1c levels and increased FINS in STZ-induced C57BL/6 mice. Male C57BL/6 mice (8 weeks old) were intraperitoneally injected with streptozotocin (STZ, 60 mg/kg/day) for 5 consecutive days. Mice in control group were injected with the same amount of citrate buffer. DHY (250 mg/kg) or carboxymethylcellulose (CMC, 0.5%) were administrated 2 weeks later, once daily by gavage for 12 weeks. (**A**) FBG level. (**B**) TG level in the serum. (**C**) HbA1c level in the plasma. (**D**) FINS level. ** *p* < 0.01 versus control, # *p* < 0.05, ## *p* < 0.01 versus STZ, n = 6.

Hyperglycemia and disordered lipid metabolism are the mainstays underlying the pathophysiologic mechanism of DCM. STZ-induced C57BL/6 mice showed significant elevation of FBG, TG and HbA1c level, accompanied by severe insulin secretion impairment as indicated by decreased FINS compared with control mice. DHY treatment reversed FBG, TG and HbA1c levels and increased FINS in STZ-administrated mice (Figure 1).

### 3.2. DHY Improved Cardiac Dysfunction in STZ-Induced Mice

**Figure 2:**
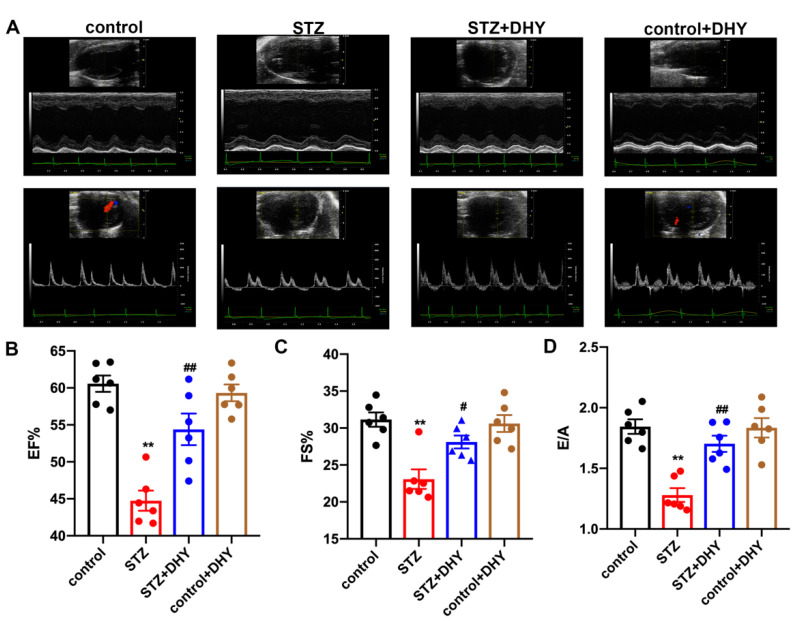
DHY improved cardiac dysfunction in STZ-induced C57BL/6 mice. (**A**) Representative echocardiographs of two-dimensional M-mode and doppler of mice. (**B**) EF. (**C**) FS. (**D**) E/A ratio. ** *p* < 0.01 versus control; # *p* < 0.05, ## *p* < 0.01 versus STZ, n = 6.

Echocardiography was performed to explore the effects of DHY on cardiac function in STZ-induced C57BL/6 mice. Mice with STZ administration displayed systolic and diastolic dysfunction as indicated with decreased EF, FS and E/A. All above three parameters were enhanced after DHY treatment, indicating that DHY improved cardiac dysfunction in STZ-induced mice (Figure 2).

### 3.3. DHY Ameliorated Myocardial Hypertrophy, Fibrosis and Injury in STZ-Induced Mice

**Figure 3:**
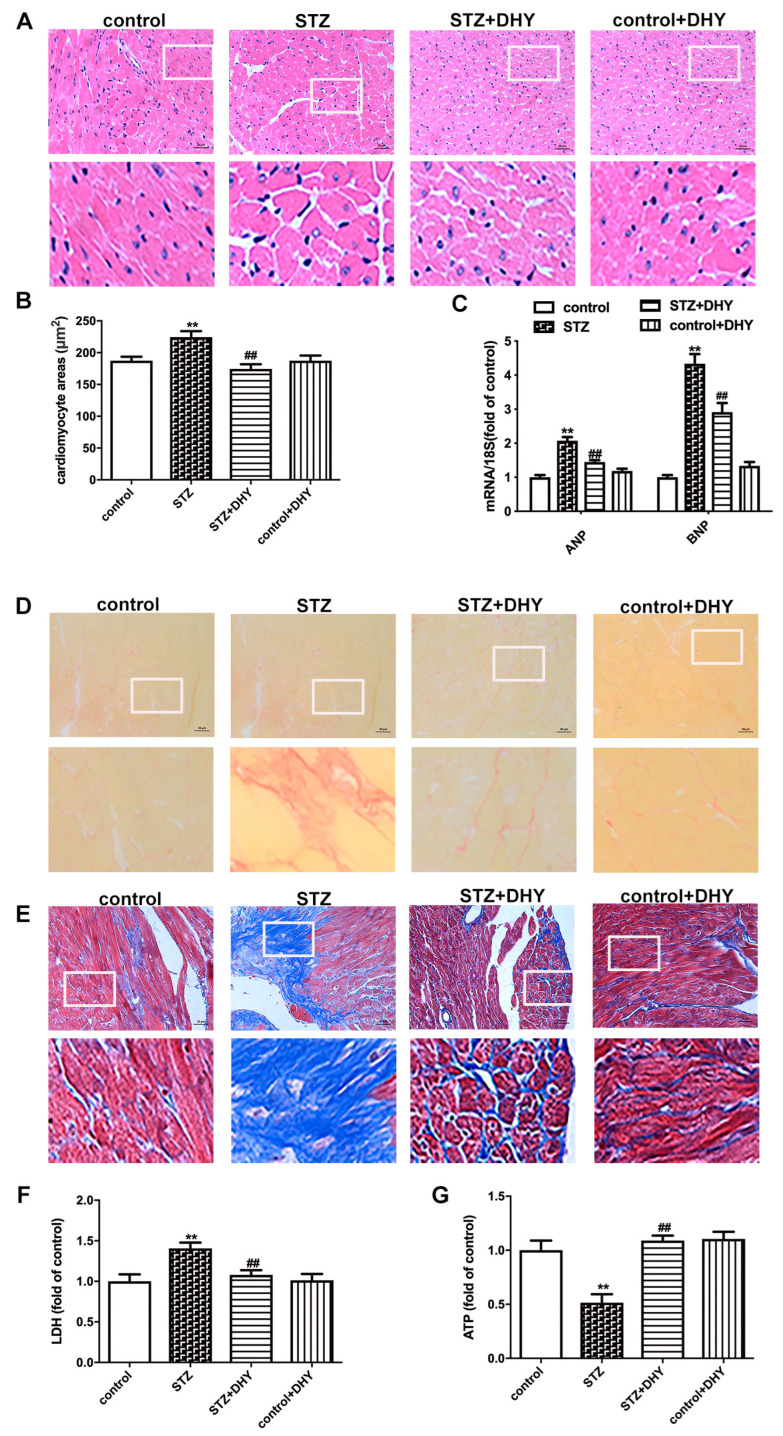
DHY ameliorated myocardial hypertrophy, fibrosis and injury in STZ-induced C57BL/6 mice. (**A**) The representative images of ventricle tissue with HE staining (bar = 20 μm). (**B**) Cardiomyocyte areas. (**C**) Expression of ANP and BNP mRNA. (**D**–**E**) The representative images of the myocardium with sirus red staining and Masson’s staining (bar = 20 μm). (**F**) LDH in the serum. (**G**) ATP level in the myocardium. ** *p* < 0.01 versus control, ## *p* < 0.01 versus STZ, n = 6.

Previous studies have revealed that both myocardial hypertrophy and fibrosis are typical characteristics of DCM. In the present study, STZ-induced C57BL/6 mice displayed enlarged cardiomyocytes and enhanced hypertrophic genes including ANP and BNP (Figure 3A–C). Sirius red staining and Masson’s staining showed more deposition of collagen in the myocardium of STZ-induced mice compared with control mice (Figure 3D,E). Furthermore, there were higher levels of serum LDH but lower level of ATP content in STZ-induced mice (Figure 3F,G), suggesting serious myocardial injury in mice with DCM. Moreover, DHY treatment ameliorated myocardial hypertrophy, fibrosis and injury in STZ-induced mice (Figure 3).

### 3.4. DHY Suppressed Oxidative Stress, Inflammasome and Necroptosis but Improved SIRT3 Expression in STZ-Induced Mice

**Figure 4:**
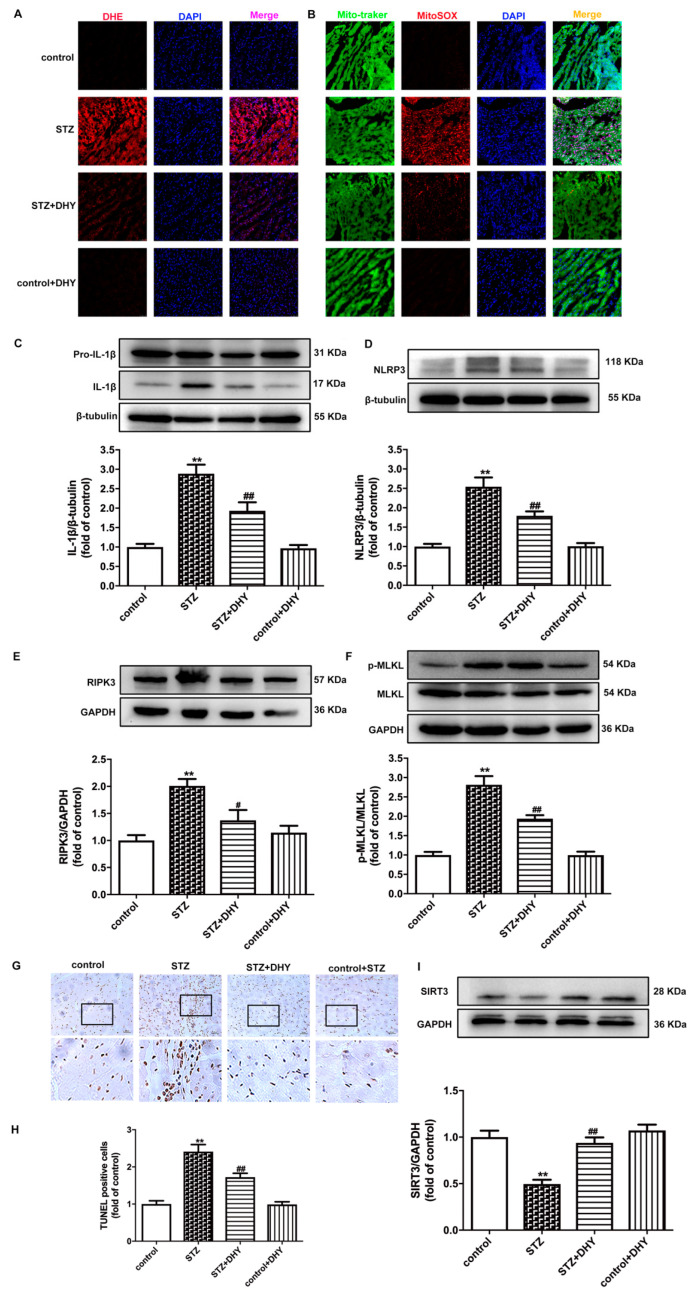
DHY suppressed oxidative stress, inflammasome and necroptosis but improved SIRT3 expression in STZ-induced C57BL/6 mice. (**A**) The representative images of myocardium with DHE staining (red) and nuclei with DAPI staining (blue) (bar = 50 μm). (**B**) The representative images of myocardium with MitoSOX staining (red), mitochondria localization with Mito-tracker staining (green) and nuclei with DAPI staining (blue) (bar = 50 μm). (**C**–**F**) Expression of IL-1β, NLRP3, RIPK3 and MLKL protein in the myocardium. (**G**,**H**) The representative images of myocardium with TUNEL staining (bar = 20 μm). TUNEL positive cells were quantified. (**I**) Expression of SIRT3 protein in the myocardium. ** *p* < 0.01 versus control; # *p* < 0.05, ## *p* < 0.01, versus STZ, n = 6.

Oxidative stress and inflammation are key roles in the pathogenesis of DCM. DHE and MitoSOX staining showed that myocardial superoxide production and mitochondrial oxidative injury were increased in STZ-induced C57BL/6 mice. Expression of IL-1β and NLRP3 were enhanced in the myocardium of STZ-induced mice. However, the above parameters were reversed after DHY treatment, indicating that DHY suppressed oxidative stress and inflammation in STZ-induced mice (Figure 4A–D).

Excessive oxidative stress and inflammation are closely related to necroptosis. RIPK3 and MLKL phosphorylation are indicators of necroptosis. In addition, apoptosis is also a vital manifestation of necroptosis. Compared with control mice, RIPK3 expression, MLKL phosphorylation and TUNEL-positive cell numbers in the myocardium of STZ-induced mice were significantly up-regulated, indicating promoted necroptosis in the myocardium of diabetic mice. However, DHY treatment significantly inhibited necroptosis in the STZ-induced mice (Figure 4E–H).

Due to the possible relation between SIRT3 and necroptosis in DCM [14], our study further verified that SIRT3 expression was obviously decreased in the myocardium of STZ-induced mice, which was restored after DHY treatment (Figure 4I).

### 3.5. DHY Attenuated Cell Damage but Improved SIRT3 Expression in Cardiomyocytes with High Glucose Stimulation

**Figure 5:**
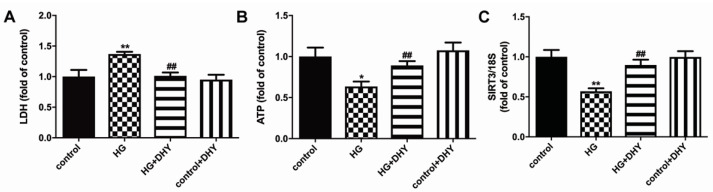
DHY attenuated cell damage but improved SIRT3 expression in cardiomyocytes with high glucose stimulation. (**A**) LDH in the culture medium. (**B**) ATP level in the cardiomyocytes. (**C**) Expression of SIRT3 mRNA in the cardiomyocytes. * *p* < 0.05, ** *p* < 0.01 versus control; ## *p* < 0.01 versus HG, n = 6.

DHY decreased LDH release but increased ATP level in high glucose-stimulated cardiomyocytes, indicating that DHY attenuated cell damage in cardiomyocytes with high glucose stimulation (Figure 5A,B). In addition, SIRT3 mRNA expression was inhibited in cardiomyocytes with high glucose stimulation, which was improved by DHY treatment (Figure 5C).

### 3.6. DHY Inhibited Oxidative Stress, Inflammasome and Necroptosis in Cardiomyocytes with High Glucose Stimulation

**Figure 6:**
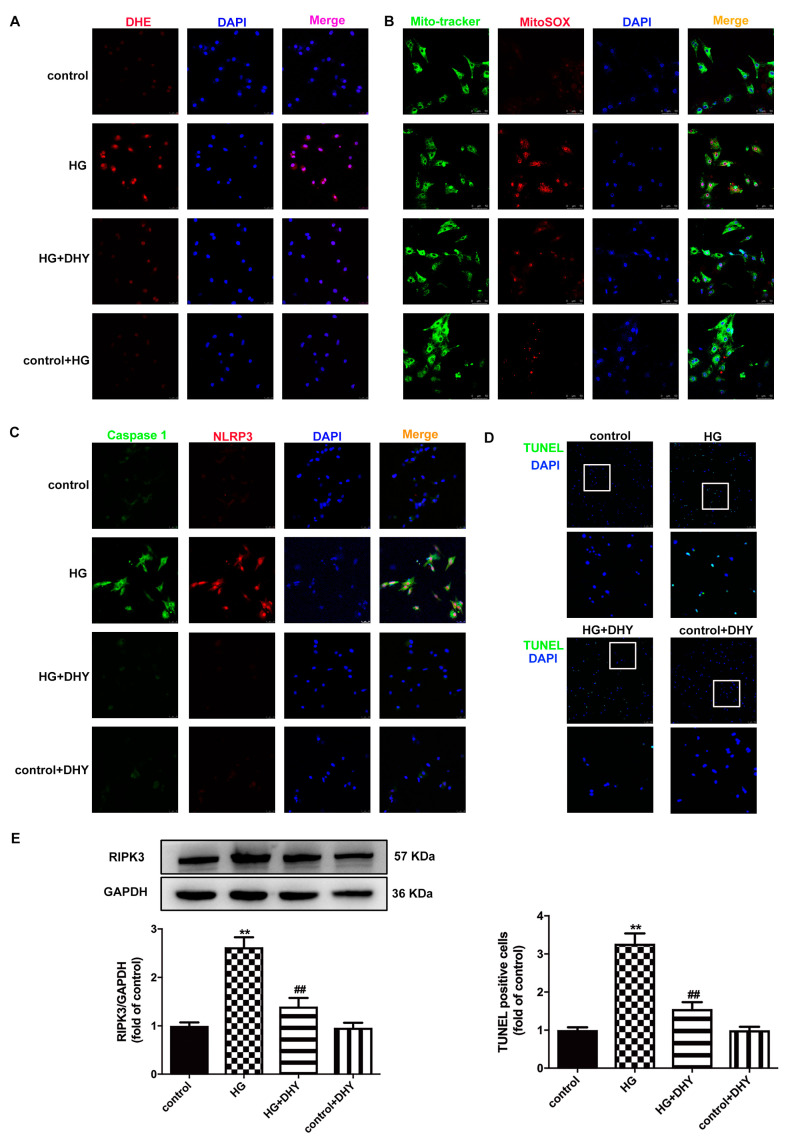
DHY inhibited oxidative stress, inflammasome and necroptosis in cardiomyocytes with high glucose stimulation. (**A**) The representative images of cardiomyocyte with DHE staining (red) and nuclei with DAPI staining (blue) (bar = 25 μm). (**B**) The representative images of cardiomyocyte with MitoSOX staining (red), mitochondria localization with Mito-tracker staining (green) and nuclei with DAPI staining (blue) (bar = 50 μm). (**C**) Caspase 1 was immunofluorescence stained with Alexa Fluor 488-conjugated IgG (green), NLRP3 with Cy3-conjugated IgG (red), and nuclei with DAPI staining (blue) (bar = 25 μm). (**D**) The representative images of cardiomyocyte with TUNEL staining (green), nuclei with DAPI staining (blue) (bar = 100 μm). TUNEL-positive cells were quantified. (**E**) Expression of RIPK3 protein. ** *p* < 0.01 versus control, ## *p* < 0.01 versus HG, n = 6.

DHY alleviated fluorescence intensity of DHE and MitoSOX staining in high glucose-stimulated cardiomyocytes, indicating that DHY inhibited oxidative stress in cardiomyocytes with high glucose stimulation (Figure 6A,B). Furthermore, DHY suppressed the expression of caspase 1 and NLRP3 and reduced TUNEL-positive cells and RIPK3 expression in HG-stimulated cardiomyocytes (Figure 6C–E). Taken together, DHY inhibited oxidative stress, inflammasome and necroptosis in cardiomyocytes with high glucose stimulation.

### 3.7. DHY Failed to Alleviate Diabetic Cardiomyopathy in STZ-Induced SIRT3-KO Mice

**Figure 7:**
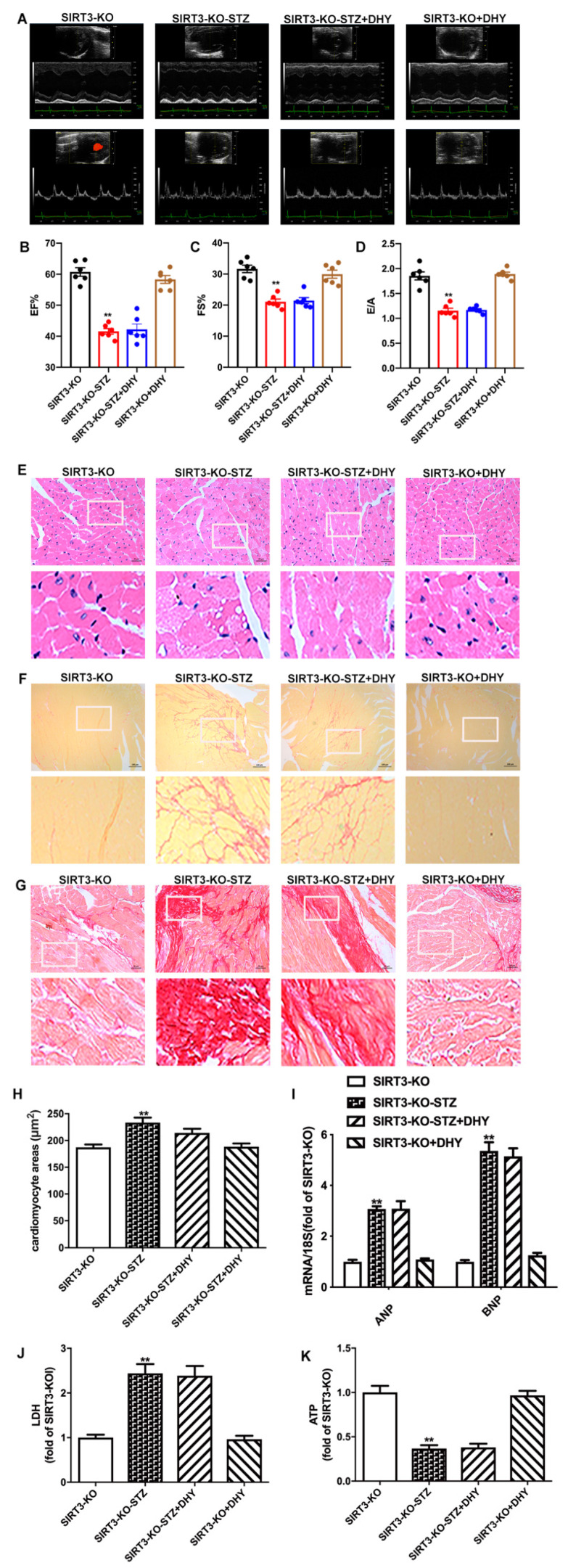
DHY failed to alleviate diabetic cardiomyopathy in STZ-induced SIRT3-KO mice. Male 8-week-old SIRT3-KO mice were injected with STZ (60 mg/kg/day) for 5 consecutive days. Mice in control group were injected the same amount of citrate buffer. DHY (250 mg/kg) or carboxymethylcellulose (CMC, 0.5%) were administrated 2 weeks later, once daily by gavage for 12 weeks. (**A**) Representative echocardiographs of two-dimensional M-mode and Doppler of mice. (**B**) EF. (**C**) FS. (**D**) E/A ratio. (**E**–**G**) The representative images of the myocardium with HE staining (bar = 100 μm), sirus red staining (bar = 100 μm) and Masson’s staining (bar = 20 μm). (**H**) Cardiomyocyte areas. (**I**) Expression of ANP and BNP mRNA. (**J**) LDH in the serum. (**K**) ATP level in the myocardium. ** *p* < 0.01 versus SIRT3-KO.

Next, SIRT3-KO mice were used to investigate the possible critical role of SIRT3 on the effects of DHY against DCM. Our results showed that there was no significant improvement of cardiac function, myocardial hypertrophy, fibrosis or myocardial injury in STZ-induced SIRT3-KO mice after DHY treatment (Figure 7). It indicated that DHY protected against DCM in a SIRT3 dependent manner.

## 4. Discussion

A long-term diabetic state is prone to inducing several pathological changes related to insulin deficiency and is also regarded as a main cause of DCM. Previous studies have demonstrated that STZ-induced diabetic mice showed impaired cardiac function, serious myocardial hypertrophy and fibrosis [14,21]. Nonetheless, there is no consensus about the clinical diagnosis of DCM, and no effective treatment. Although multiple research groups have tried to seek novel agents against DCM, this is also a pending topic. Previous studies and clinical efficacy investigations have verified that DHY attenuated alloxan-caused pancreatic β-cells damage via the improvement of anti-oxidant effects [22]. DHY also improved glucose uptake in adipocytes [23]. Accordingly, the hypoglycemic effect of DHY in STZ-induced diabetes of our present research may be also attributed to the enhancement of anti-oxidative ability and glucose uptake. In the present study, DHY reversed FBG, TG, HbA1c level and increased FINS in STZ-induced mice. We found that DHY alleviated STZ-induced cardiac dysfunction, myocardial hypertrophy and cardiac fibrosis, inhibited STZ-induced oxidative stress, inflammation and necroptosis in vitro and in vivo. In addition, we noted that SIRT3 knockout abolished all protective effects of DHY against DCM. Our results indicated that DHY exerted antioxidant, anti-inflammatory and anti-necroptotic effects on DCM depending on SIRT3 activation.

DHY has been widely used for decades by virtue of numerous beneficial effects including metabolic regulation, free radical scavenging, antioxidant effects, anti-inflammation, neuroprotection and cardiovascular protection [2,24,25,26]. Our previous studies have showed that DHY attenuated Ang II-induced rat cardiomyocyte hypertrophy and cardiac fibroblasts proliferation, inhibited myocardial hypertrophy after transverse aortic constriction and improved endothelial dysfunction in diabetic mice [4,5,6,7]. In our study, DHY alleviated FBG and improved cardiac function with beneficial effects on EF, FS and E/A ratio. Furthermore, DHY decreased cardiomyocyte areas, inhibited expression of hypertrophic gene ANP and BNP and ameliorated collagen deposition in the myocardium. DHY treatment also alleviated high glucose-induced cardiomyocyte injury. Taken together, these data exhibited solid evidence of the cardioprotective roles of DHY on DCM.

Oxidative stress and inflammation are involved in DCM [13,27]. In our study, DHY alleviated oxidative stress, manifesting as the decrease in superoxide production and mitochondrial superoxide levels in STZ-induced mice or high glucose-induced cardiomyocytes. DHY suppressed NLRP3 expression and IL-1β maturity, thereby depressing inflammation in STZ-induced mice and high glucose-induced cardiomyocytes. In addition, several studies have focused on the relationship between necroptosis and DCM. Previous studies demonstrated that DHY treatment improved cardiac function by down-regulation of high mobility group box-1 (HMGB1) and phospho-NF-κB p65 expressions in the myocardium. In another study, DHY prevented DCM via miR-3a suppression by activating autophagy. DHY treatment also inhibited cardiac apoptosis in diabetic mice [28,29,30]. However, the possible beneficial effects of DHY on necroptosis during DCM have not been fully elucidated. Our present study confirmed the involvement of necroptosis in the preventive effects against DCM by DHY, which is a novel mechanism for DHY. One report described that exposure to high glucose promoted the expression of necroptosis related genes RIPK1, RIPK3 and MLKL in H9C2 cells. The specific inhibitor of necroptosis Necrostain-1 attenuated high glucose-induced up-regulation of RIPK1, RIPK3 and MLKL to alleviate cell damage [31]. Our previous study showed aggravated DCM in STZ-stimulated mice, as well as in (Lepr) KO/KO (db/db) mice [21]. Here, we found DHY treatment decreased RIPK3 expression, MLKL phosphorylation and TUNEL-positive cell numbers in STZ-induced mice and high glucose-induced cardiomyocytes, indicating that DHY inhibited necroptosis in DCM. In addition, caspase 3 cleavage, bax and bcl expression are all cell death markers. Our previous studies demonstrated that cleaved caspase 3 expression was increased in STZ-induced mice and advanced glycation end products (AGEs) stimulated cardiomyocytes [32]. Hyperglycemia may influence bax and bcl expression, which are also vital for DCM. Therefore, the influence of DHY on the indexes is helpful to understand the protective mechanism, which needs further studies in future.

SIRT3 is a histone deacetylase to regulate cardiac cell growth, energy metabolism, reactive oxygen species production, inflammation and cell death, and, thus, it plays a vital role in cardiovascular diseases [18,33,34,35]. We have demonstrated the protective role of SIRT3 in cholesterol accumulation and foam cell formation of macrophages with ox-LDL stimulation [8]. We also demonstrated the critical role of SIRT3 in the alleviation of hypoxia-induced cardiac fibroblasts proliferation by hydrogen sulfide [36]. Wei et al. also reported that DHY ameliorated cardiac ischemia/reperfusion injury through SIRT3 activation [37]. In the present study, DHY administration increased SIRT3 expression to protect STZ-induced mice and high glucose-induced cardiomyocytes. Moreover, the protective effects of DHY on DCM were abolished by SIRT3 knockout, indicating that DHY alleviated DCM through SIRT3 activation. Previous study demonstrated that the improved activator protein 1 (AP-1) binding activity with SIRT3 promoter contributed to the enhanced SIRT3 expression in vascular endothelial cells [38]. Peroxisome proliferator-activated receptor-gamma coactivator 1 α (PGC-1α) is reported to positively regulate the expression of SIRT3 in DCM, ischemia-induced oxidative stress and mitochondrial biogenesis [39,40,41]. In short, DHY may increase the binding activity of AP-1, PGC-1α, or some other factors with SIRT3 promoter, then increasing SIRT3 expression and ultimately protecting against DCM. In addition, our previous investigations demonstrated that superoxide dismutase 2 (SOD2) as a down-stream target of SIRT3 participated in the protective effect of endothelial dysfunction in diabetic mice by DHY [7]. We also confirmed that both forkhead box protein 3a (FOXO3a) and SOD2 are down-stream targets to regulate mitochondrial function and oxidative stress in myocardial hypertrophy [4,42]. However, whether similar molecular targets after SIRT3 activation by DHY contribute to prevent cardiomyocyte apoptosis and/or necroptosis needs further studies in future.

## 5. Conclusions

The present data indicated that DHY improved cardiac dysfunction, ameliorated myocardial hypertrophy, fibrosis and injury and suppressed oxidative stress, inflammation and necroptosis via SIRT3 activation in STZ-induced diabetic mice, suggesting DHY may serve as a candidate agent to attenuate diabetic cardiomyopathy.

## Data Availability

All the data are available within the article.
